# PPM1D modulates hematopoietic cell fitness and response to DNA damage and is a therapeutic target in myeloid malignancy

**DOI:** 10.1182/blood.2023020331

**Published:** 2023-08-20

**Authors:** Peter G. Miller, Adam S. Sperling, Christina Mayerhofer, Marie E. McConkey, Jana M. Ellegast, Carmen Da Silva, Drew N. Cohen, Chuqi Wang, Azeem Sharda, Ni Yan, Subha Saha, Cameron Schluter, Ilexa Schechter, Mikołaj Słabicki, Brittany Sandoval, Josephine Kahn, Steffen Boettcher, Christopher J. Gibson, David T. Scadden, Kimberly Stegmaier, Shruti Bhatt, R. Coleman Lindsley, Benjamin L. Ebert

**Affiliations:** 1 Center for Cancer Research, Massachusetts General Hospital, Boston, MA; 2 Center for Regenerative Medicine, Massachusetts General Hospital, Boston, MA; 3 Harvard Medical School, Boston, MA; 4 Broad Institute of MIT and Harvard, Cambridge, MA; 5 Department of Medical Oncology, Dana-Farber Cancer Institute, Boston, MA; 6 Division of Hematology, Department of Medicine, Brigham and Women's Hospital, Boston, MA; 7 Harvard Stem Cell Institute, Harvard University, Cambridge, MA; 8 Department of Pediatric Oncology, Dana-Farber Cancer Institute, Boston, MA; 9 National University of Singapore, Singapore; 10 Department of Medical Oncology and Hematology, University of Zurich and University Hospital Zurich, Zurich, Switzerland; 11 Department of Medicine, Brigham and Women's Hospital, Boston, MA; 12 Comprehensive Cancer Center Zurich, Zurich, Switzerland; 13 Department of Stem Cell and Regenerative Biology, Harvard University, Boston, MA; 14 Ludwig Center at Harvard, Boston, MA; 15 Howard Hughes Medical Institute, Bethesda, MD

## Abstract

*PPM1D* encodes a phosphatase that is recurrently activated across cancer, most notably in therapy-related myeloid neoplasms. However, the function of PPM1D in hematopoiesis and its contribution to tumor cell growth remain incompletely understood. Using conditional mouse models, we uncover a central role for Ppm1d in hematopoiesis and validate its potential as a therapeutic target. We find that Ppm1d regulates the competitive fitness and self-renewal of hematopoietic stem cells (HSCs) with and without exogenous genotoxic stresses. We also show that although Ppm1d activation confers cellular resistance to cytotoxic therapy, it does so to a lesser degree than p53 loss, informing the clonal competition phenotypes often observed in human studies. Notably, loss of Ppm1d sensitizes leukemias to cytotoxic therapies in vitro and in vivo, even in the absence of a *Ppm1d* mutation. Vulnerability to PPM1D inhibition is observed across many cancer types and dependent on p53 activity. Importantly, organism-wide loss of Ppm1d in adult mice is well tolerated, supporting the tolerability of pharmacologically targeting PPM1D. Our data link *PPM1D* gain-of-function mutations to the clonal expansion of HSCs, inform human genetic observations, and support the therapeutic targeting of PPM1D in cancer.

## Key Points

• Ppm1d activity is a key regulator of hematopoietic cell fitness in the absence and presence of exogenous genotoxic stresses.
• Inhibition of Ppm1d sensitizes malignant cells to cytotoxic therapies and is dependent of p53 activity.

## Visual Abstract

**Figure:**
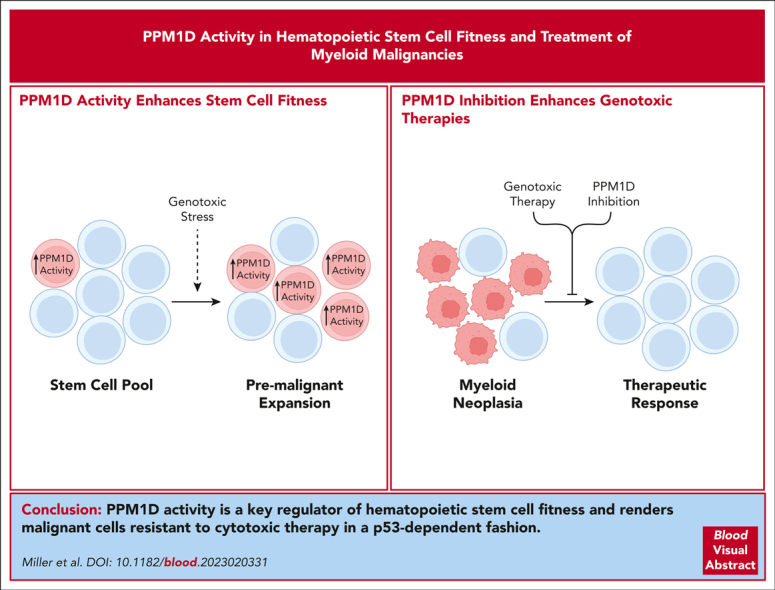

## Teaser

Miller et al examined the role of increased activity of protein phosphatase Mg2^+^/Mn2^+^ dependent 1D (PPM1D), a negative regulator of *TP53* and the DNA damage repair pathway, on chemotherapeutic resistance of hematopoietic stem cells (HSCs). The authors’ data indicate that PPM1D is a crucial regulator of HSC fitness and that its absence or pharmacological inhibition leads to greater sensitivity of malignant cells to chemotherapy, including in acute myeloid leukemia (AML) xenografts. These findings will prompt further exploration of PPM1D as a therapeutic target in AML.

## Introduction

The DNA damage response (DDR) orchestrates the cellular reaction to endogenous and exogenous genotoxic stresses. Numerous cellular programs are regulated by the DDR, including cell cycle arrest, DNA repair, senescence, and apoptosis. p53 is activated upon DNA damage and serves as a critical node in the DDR, and there are many genetic alterations across cancer types that result in loss of p53 activity, including mutation and/or deletion of the *TP53* locus. The study of somatic mutations in blood cells of individuals exposed to cytotoxic therapy has demonstrated that genes involved in the DDR are recurrently mutated, largely restricted to *PPM1D*, *TP53*, *ATM*, *CHEK2*, and *SRCAP*.^1^, ^2^, ^3^, ^4^ *PPM1D* and *TP53* are by far the most commonly mutated among this group, suggesting that both play a central role in the response to genotoxic stress in hematopoietic stem cells (HSCs).

*PPM1D* encodes for a serine/threonine phosphatase that is transcriptionally activated by p53 and negatively regulates the DDR and p53 signaling via dephosphorylation of numerous substrates upstream of p53, downstream of p53, and p53 itself. Consistent with its function as a suppressor of the DDR/P53, *PPM1D* is recurrently activated in cancer via amplifications and activating mutations.^5^ We and others have shown that *PPM1D* is recurrently mutated in clonal hematopoiesis and myeloid cancers, particularly in patients who have received cytotoxic therapy in the form of chemotherapy or radiation.^1^, ^2^, ^3^^,^^6^^,^^7^ These mutations truncate the C-terminus of the protein, resulting in the loss of a proteasomal degradation signal and elevated intracellular levels of the enzymatically active protein. When this occurs, activation of p53 and other members of the DDR are suppressed, resulting in selective outgrowth of cells carrying *PPM1D* mutations in the presence of cytotoxic agents.

Given the frequency of *PPM1D* alterations observed across many oncologic contexts and its role as a regulator of p53 activation and the DDR, PPM1D has emerged as a potential drug target across numerous indications. To date, a germ line knockout of *Ppm1d* and a germ line introduction of a truncating mutation in *Ppm1d* have been generated and characterized.^6^^,^^8^ To examine the consequences of *Ppm1d* truncation and inactivation selectively in hematopoietic cells, we generated conditional *Ppm1d* knockout and conditional *Ppm1d* truncating mutant knock-in mouse strains. Using these models to examine the role of Ppm1d in HSC biology and the therapy of myeloid malignancies, we found that despite being an important regulator of HSC fitness, PPM1D is also a therapeutic target to augment the efficacy of cytotoxic chemotherapy and radiation.

## Methods

### Generation of transgenic mouse models and competitive transplants

**Figure 1.**
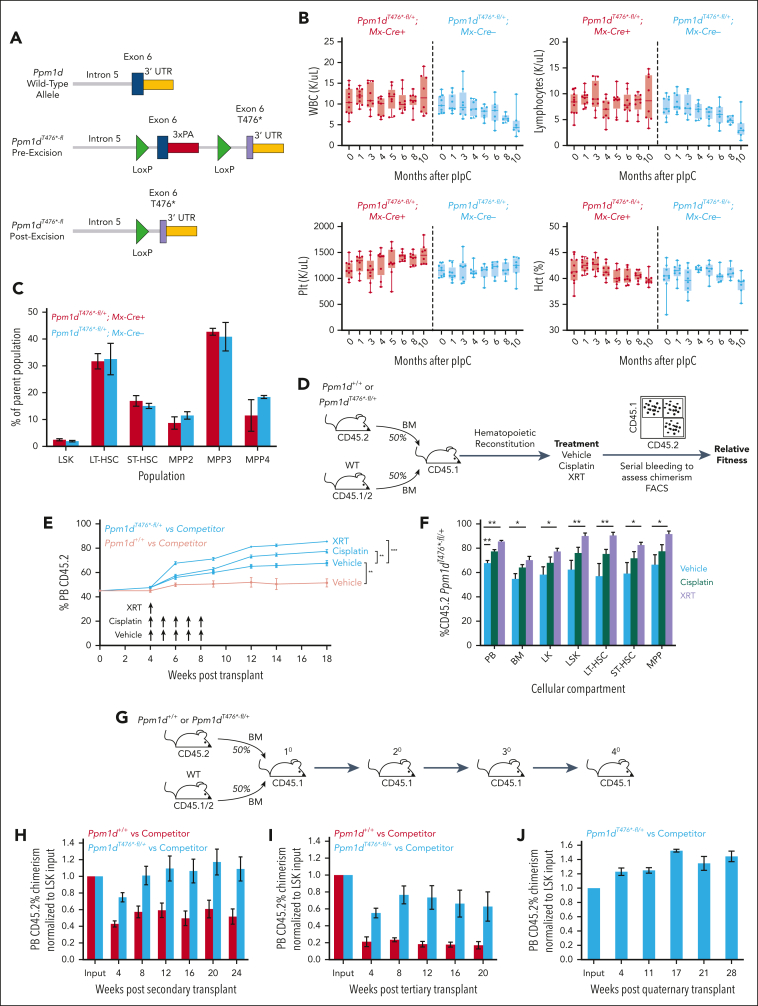
**Truncating mutations in *Ppm1d* enhance HSC fitness.** (A) Schematic of engineered locus in *Ppm1d*^*T476*^^*∗*^^*-fl*^ mice. (B) Peripheral blood white blood cell (WBC), lymphocyte, platelet (Plt) counts, and hematocrit (Hct) of *Ppm1d*^*T476*^^*∗*^^*-fl/+*^;*MxCre*^*+*^ or *Ppm1d*^*T476*^^*∗*^^*-fl/+*^;*MxCre*^*−*^ mice treated with pIpC at age 10 weeks. (C) Bone marrow stem cell analysis of *Ppm1d*^*T476*^^*∗*^^*-fl/+*^;*MxCre*^*+*^ or *Ppm1d*^*T476*^^*∗*^^*-fl/+*^;*MxCre*^*−*^ mice approximately 1 year after pIpC treatment. (D) Schematic of competition experiment between *Ppm1d*^*T476*^^*∗*^^*-fl/+*^;*Vav-Cre*^+^;*Cd45.2* or *Ppm1d*^*+/+*^;*Vav-Cre*^+^;*Cd45.2* and wild-type (WT) *Vav-Cre*^*+*^;*Cd45.1/2* control bone marrow cells transplanted into lethally irradiated *Cd45*.1 recipients. Cisplatin was dosed intraperitoneally at 4 mg/kg and sublethal irradiation was dosed at 2.5 Gy. (E-F) Peripheral blood (E) and bone marrow (F) CD45.2 chimerism of recipient mice from *Ppm1d*^*T476*^^*∗*^^*-fl/+*^;*Vav-Cre*^*+*^;*Cd45.2* and WT *Cd45.1/2* competition experiment outlined in panel D. (G) Schematic of serial transplantation of the bone marrow from the vehicle control mice outline in panel D. (H-J) Peripheral blood Cd45.2 chimerism of secondary (H), tertiary (I), and quaternary (J) mice serially transplanted with *Ppm1d*^*+/+*^;*Vav-Cre*^+^;*Cd45.2* and WT *Vav-Cre*^*+*^;*Cd45.1/2* (gray) or *Ppm1d*^*T476*^^*∗*^^*-fl/+*^;*Vav-Cre*^+^;*Cd45.2* and WT *Vav-Cre*^*+*^;*Cd45.1/2* (black). Note that in the quaternary transplant only *Ppm1d*^*T476*^^*∗*^^*-fl/+*^;*Vav-Cre*^+^;*Cd45.2* were present. Error bars show standard error of the mean (SEM), ∗*P* < .05, ∗∗*P* < .01, ∗∗∗*P* < .001.

**Figure 2.**
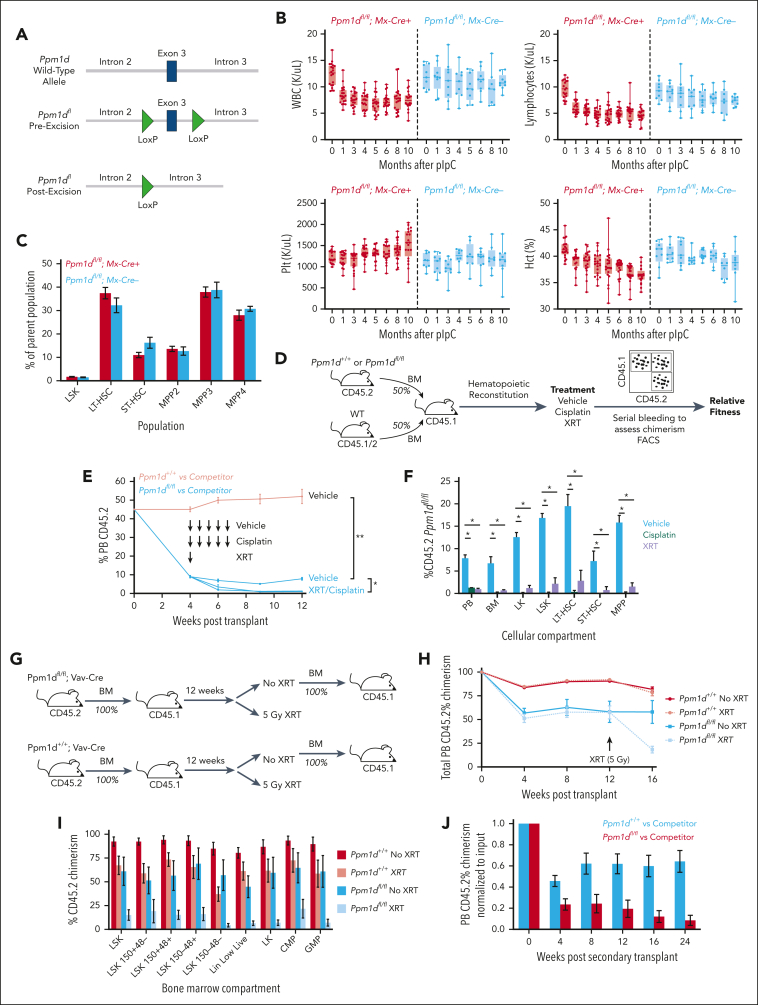
***Ppm1d* loss impairs HSC fitness.** (A) Schematic of engineered locus in *Ppm1d*^*fl/fl*^ mice (left) and genotyping polymerase chain reaction showing different allelic states (right). (B) Peripheral blood WBC, lymphocyte, Plt counts, and Hct of *Ppm1d*^*fl/fl*^;*MxCre*^*+*^ or *Ppm1d*^*fl/fl*^;*MxCre*^*−*^ mice treated with pIpC at age 10 weeks. (C) Bone marrow stem cell analysis of *Ppm1d*^*fl/fl*^;*MxCre*^*+*^ or *Ppm1d*^*fl/fl*^;*MxCre*^*−*^ mice approximately 1 year after pIpC treatment. (D) Schematic of competition experiment between *Ppm1d*^*fl/fl*^;*Vav-Cre*^+^;*Cd45.2* or *Ppm1d*^*+/+*^; *Vav-Cre*^+^;*Cd45.2* and wild-type (WT) *Vav-Cre*^*+*^;*Cd45.1/2* control bone marrow cells transplanted into lethally irradiated *Cd45*.1 recipients. Cisplatin was dosed intraperitoneally at 4 mg/kg and sublethal irradiation was dosed at 2.5 Gy. (E-F) Peripheral blood (E) and bone marrow (F) CD45.2 chimerism of recipient mice from *Ppm1d*^*fl/fl*^;*Vav-Cre*^*+*^;*Cd45.2* and WT *Cd45.1/2* competition experiment outlined in panel D. (G) Schematic of serial transplantation and irradiation experiment of *Ppm1d*^*fl/fl*^;*Vav-Cre*^+^ or *Ppm1d*^*+/+*^;*Vav-Cre*^+^ bone marrow cells. The irradiation group received 5 Gy. (H-I) Peripheral blood (H) and bone marrow (I) CD45.2 chimerism of primary transplant recipients of *Ppm1d*^*+/+*^;*Vav-Cre*^+^ (gray) and *Ppm1d*^*fl/fl*^;*Vav-Cre*^+^ (black) bone marrow cells. (J) Peripheral blood CD45.2 chimerism of secondary transplant recipients of *Ppm1d*^*+/+*^;*Vav-Cre*^+^ (gray) and *Ppm1d*^*fl/fl*^;*Vav-Cre*^+^ (black) bone marrow cells. Error bars show SEM, ∗*P* < .01, ∗∗*P* < .0001.

*Ppm1d*^*T476*^^*∗-fl/+*^ and *Ppm1d*^*fl/fl*^ mice were generated via homologous recombination by the Gene Targeting and Transgenic Facility at the Janelia Research campus at the Howard Hughes Medical Institute. The FLP recombinase target sites and neomycin cassette were removed by crossing with C57BL/6 FLP mice (Figures 1A and 2A). Competitive whole bone marrow transplants, drug exposures, and stem and progenitor analyses were performed as previously described (supplemental Methods, available on the *Blood* website).^9^ Treatments included intraperitoneal administration of normal saline vehicle weekly for 5 doses, intraperitoneal administration of cisplatin (Selleck Chemical, diluted to 4 mg/kg final in normal saline) weekly for 5 doses, or a single dose of 250 cGy radiation.

### Generation, and culture of mouse leukemia cells

c-Kit^+^ cells were isolated from the bone marrow using CD117 selection beads (Miltenyi) and transduced with MLL-AF9-GFP retrovirus.^10^ After 48 hours, the cells were transplanted into sublethally irradiated (450 cGy) Bl6.SJL *CD45.1*^*+*^ recipient mice. Primary leukemia cells were then cultured in Iscove modified Dulbecco medium supplemented with 20% fetal bovine serum, mouse stem cell factor (25 ng/μL), mouse interleukin-3 (10 ng/mL), and mouse interleukin-6 (5 ng/mL). In vitro drug treatments were subsequently performed as outlined in the supplemental Methods.

### In vivo drug treatment of mouse leukemia cells

Wild-type (WT), nonlethally irradiated mice were engrafted with 50 000 luciferase-expressing, MLL-AF9^+^ GFP^+^ primary leukemia cells as previously described.^11^ Ten days later, the leukemia burden was assessed using the in vivo imaging system (PerkinElmer). Intraperitoneal injection of cytarabine, doxorubicin, or saline and oral gavage of GSK2830371 were then performed.

### Human PDX studies

For the in vitro cell viability assays involving the 4 patient–derived xenograft (PDX) models, cells were grown in cytokine-supplemented media.^12^^,^^13^ The cells were then exposed to drugs at the indicated concentrations for 72 hours and viability was measured using the CellTiter-Glo reagent. For the dynamic BH3 profiling on PDX models, myeloblasts harvested from mouse cohorts harboring 5 PDX models (n = 3 mice per model) were exposed to GSK2830371 for 14 hours, followed by dynamic BH3 profiling to determine delta priming in response to BIM-BH3, as previously described.^14^

### Cell line studies

The CRISPR/Cas9 screen was performed on previously described engineered K562 using a custom library of small guide RNAs (sgRNAs), encoded by lentivirus obtained from the Broad Institute (supplemental Methods).^15^^,^^16^ After puromycin selection, the cells were grown for 3 weeks in dimethyl sulfoxide, daunorubicin, or GSK2830371, and then the representation of each sgRNA was quantified as previously described.^17^^,^^18^ Cell viability assays were performed using CellTiter-Glo (Promega) after 3 days of exposure to drug. The drug screen to assess for the effects of GSK2830371 on sensitivity of 750 DNA-barcoded cell lines to daunorubicin was performed using the PRISM platform, as previously described.^19^^,^^20^ Data from The Cancer Dependency Map at the Broad Institute of MIT and Harvard were accessed via the web portal www.depmap.org/portal/.^21^

### Statistical analysis

The Mann-Whitney *U* test or the Student *t* test was used to test the statistical difference between continuous variables. All statistical analyses were performed using the Prism software package (Graphpad, v9.5.0).

## Results

### *Ppm1d* truncating mutations enhance the competitive fitness of hematopoietic cells

To examine the role of Ppm1d activity in specific tissues, including the hematopoietic system in which *PPM1D* is recurrently mutated in humans, we generated a genetically engineered mouse model of Ppm1d activation via conditional introduction of a C-terminal truncating mutation. LoxP sites were placed on both sides of the endogenous exon 6 of *Ppm1d*, and a truncated version of exon 6 at threonine 476 (T476^∗^) was introduced distal to the 3′ LoxP site, reflecting the somatic *PPM1D* truncation mutations commonly observed in humans.^1^, ^2^, ^3^^,^^6^^,^^22^ After exposure to Cre-recombinase, the WT exon 6 was removed, resulting in a truncated form of the protein (Figure 1A). A heterozygous allele state in hematopoietic cells (*Ppm1d*^*T476*^^*∗*^^*-fl*^^*/+*^), as seen in humans, was achieved by crossing these animals to either *Vav-Cre* mice, in which hematopoietic cells express Cre-recombinase starting during development, or *Mx-Cre* mice, in which Cre-recombinase is expressed in hematopoietic cells after exposure to polyinosinic:polycytidylic acid (pIpC) (supplemental Figure 1A).

To assess the effects of the truncating mutation during development, we analyzed 3-month-old *Ppm1d*^*T476∗-fl/+*^*;Vav-Cre* or WT littermate controls and found no significant difference in peripheral blood counts or stem and progenitor cell composition compared with WT littermate controls (supplemental Figure 1B-D). Similarly, *Ppm1d*^*T476∗-fl/+*^*;Mx-Cre* or WT littermate controls treated with pIpC at the age 10 weeks showed no significant differences in the peripheral blood or bone marrow composition over a 10 month observation period (Figure 1B-C).

Given the role of Ppm1d in response to DNA damage, we performed competitive bone marrow transplantation using the *Vav-Cre* model of *Ppm1d*^*T476∗-fl/+*^^*/+*^ or *Ppm1d*^*+/+*^ cells with WT competitor cells. Recipient mice were treated with vehicle, weekly cisplatin (4 mg/kg), or radiation (2.5 Gy), a dose that selects for HSCs carrying *Trp53* mutations (Figure 1D).^15^ In this competitive setting, peripheral blood and stem cell analyses revealed a significant advantage for *PPpm1d*^*T476∗-fl/+*^^*/+*^ cells with transplant alone, with a further advantage after exposure to cisplatin and radiation (Figure 1E-F; supplemental Figure 1E-F). Under the proliferative stress of serial transplantation, *Ppm1d*^*T476∗-fl/+*^^*/+*^ cells maintained an advantage relative to competitor cells that persisted through quaternary transplants. In contrast, WT cells became gradually depleted in secondary and tertiary transplants and were incapable of repopulating mouse hematopoiesis on quaternary transplantation, demonstrating that Ppm1d activation enhances serial transplantability of HSCs (Figure 1H-J). In aggregate, these studies show that conditional activation of Ppm1d provides a competitive advantage to hematopoietic stem and progenitor cells in competitive transplantation assays, in serial transplantation studies, and in response to DNA damaging agents.

### *Ppm1d* loss impairs the competitive fitness of hematopoietic cells and ability to serially transplant

Therapeutic targeting of PPM1D requires an understanding of the biological implications of PPM1D inactivation on normal and malignant cells. We therefore generated a conditional *Ppm1d* knockout model in which exon 3 of *Ppm1d*, which encodes for a core part of the protein, was flanked by LoxP sites, resulting in excision after exposure to Cre-recombinase (Figure 2A; supplemental Figure 2A). At age 3 months, *Ppm1d*^*fl/fl*^*;Vav-Cre* had no observable hematopoietic differences compared with WT littermate controls (supplemental Figure 2B-D). Compared with WT littermate controls, *Ppm1d*^*fl/fl*^*;Mx-Cre* mice treated with pIpC at age 10 weeks showed a decrease in peripheral blood B cells, a phenotype observed in the germ line knockout model, without other significant differences peripheral blood or bone marrow composition over a 10 month period (Figure 2B-C).^23^

Using the *Vav-Cre* model, we performed competitive bone marrow transplantation of *Ppm1d*^*fl/fl*^*;* or *Ppm1d*^*+/+*^ cells with WT competitor cells. Recipient mice were treated with vehicle, weekly cisplatin (4 mg/kg), or radiation (2.5 Gy) (Figure 2D).^15^ Loss of *Ppm1d* resulted in a significant competitive disadvantage, which was worsened after exposure to either cytotoxic stress (Figure 2E-F). To further interrogate the HSC defect in cells lacking *Ppm1d*, we performed a transplant with either 100% *Ppm1d*^*+/+*^ or *Ppm1d*^*fl/fl*^ bone marrow cells into lethally irradiated recipient mice (Figure 2G). Even in this setting, the *Ppm1d*^*fl/fl*^ cells did not achieve full chimerism, with evidence of partial reconstitution by recipient cells (Figure 2H). Moreover, sublethal irradiation (5 Gy) administered 12 weeks after transplant resulted in a further selective disadvantage of the *Ppm1d*^*fl/fl*^ cells compared with the WT competitor in the peripheral blood and stem cell compartments (Figure 2H-I). Finally, we performed secondary transplants of whole bone marrow from the *Ppm1d*^*fl/fl*^ or *Ppm1d*^*+/+*^ primary recipients and found that *Ppm1d*^*fl/fl*^ cells were lost over the subsequent 24 weeks, with very few remaining at the time of harvest (Figure 2J). These data demonstrate the Ppm1d is required for HSC fitness and self-renewal and are consistent with the opposite phenotype observed with *Ppm1d* truncating mutations.

Next, we studied whether the competitive fitness disadvantage of *Ppm1d*^*fl/fl*^ cells is mediated by p53. Conditional introduction of a heterozygous R172H mutation in *Trp53* has previously been shown to drive a competitive advantage in HSCs after a single, 2.5 Gy dose of radiation.^15^ CD45.2 bone marrow from either *Ppm1d*^*+/+*^;*Trp53*^*+/+*^, *Ppm1d*^*fl/fl*^;*Trp53*^*+/+*^, or *Ppm1d*^*fl/fl*^;*Trp53*^*R172H/+*^ were transplanted in a 20:80 ratio with WT, CD45.1/2 bone marrow into CD45.1 recipients. Four weeks after engraftment, half of the mice from each group were subjected to 2.5 Gy of irradiation. Over the subsequent 6 months, we observed that the competitive defect of *Ppm1d* loss in the setting of a competitive repopulation assay, with or without irradiation, was completely rescued by the presence of a *Trp53* R172H mutation. These data suggest that the observed phenotype of impaired HSC competitive fitness upon *Ppm1d* loss is dependent on p53 (supplemental Figure 2E-G).

Ppm1d has been shown to negatively regulate NF-κb, a pathway that itself influences stem cell survival in the face of inflammation. We therefore hypothesized that Ppm1d would influence the competitive fitness of hematopoietic cells after the inflammatory stress of serial pIpC, as has been previously reported.^24^ Cohorts of 1:1 mice that underwent competitive transplantation were subjected to 10 mg/kg of pIpC administered every other day for 7 doses. In contrast to the fitness changes observed with exposure to cisplatin and radiation, we did not observe any significant competitive fitness advantage or disadvantage for either *Ppm1d*^*T476*^^*∗*^^*-fl*^^*/+*^ or *Ppm1d*^*fl/fl*^ relative to WT cells in the weeks after pIpC treatment, suggesting that Ppm1d does not influence the hematologic response to this specific inflammatory exposure (supplemental Figure 2H-I).

To model the effects of a systemically administered inhibitor of Ppm1d, we crossed the *Ppm1d*^*fl/fl*^ mice with the Cre-ER^T2^, in which Cre-recombinase is expressed ubiquitously after exposure to tamoxifen.^25^ *Ppm1d*^*fl/fl*^ mice or *Ppm1d*^*+/+*^ mice were treated with tamoxifen at age 8 weeks and then monitored for 7 months (supplemental Figure 3A). Aside from the previously noted lower lymphocyte counts in the knockout animals, we observed no other hematologic or nonhematologic phenotype (supplemental Figure 3B). There was no significant difference in peripheral blood counts, stem cell composition, survival, or histologic evidence of end organ damage between the genotypes after a single or 2 sequential doses of sublethal irradiation (5 Gy) (supplemental Figure 3C-E). These data suggests that acute, organism-wide deletion of *Ppm1d* in adult animals is tolerated, even in the presence of a DNA damaging insult.^8^

### *TP53* loss confers a more pronounced selective advantage than *PPM1D* activation after genotoxic exposure

**Figure 3.**
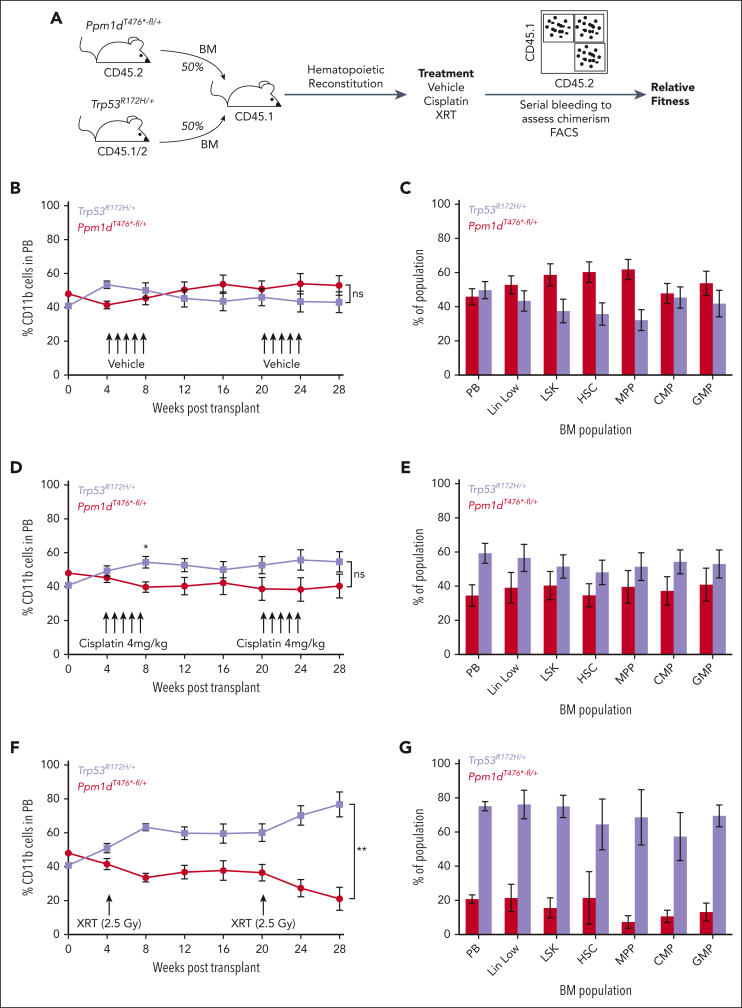
**HSCs with dominant negative mutations in *Trp53* outcompete those with *Ppm1d* truncating mutations after radiation.** (A) Schematic of competition experiment between *Trp53*^*R172H/+*^;*Vav-Cre*^+^;*Cd45.1/2* and *Ppm1d*^*T476*^^*^∗^*^^*-fl/+*^;*Vav-Cre*^+^;*Cd45.2* bone marrow cells transplanted into lethally irradiated *Cd45*.1 recipients. Cisplatin was dosed intraperitoneally at 4 mg/kg and sublethal irradiation was dosed at 2.5 Gy. (B-C) Peripheral blood CD11b^+^ (B) or bone marrow (C) CD45.2 chimerism in vehicle treated recipient mice. (D-E) Peripheral blood CD11b^+^ (D) or bone marrow (E) CD45.2 chimerism in cisplatin treated recipient mice. (F-G) Peripheral blood CD11b^+^ (F) or bone marrow (G) CD45.2 chimerism in radiation (XRT) treated recipient mice. Error bars show SEM, ∗*P* < .01, ∗∗*P* < .0001, ns, not significant.

*PPM1D* and *TP53* are the most commonly mutated DDR genes in hematopoietic cells after cytotoxic exposure and are often found in distinct clones, but the relative ability of these alterations to suppress the DDR is unknown.^1^^,^^6^^,^^26^ We therefore directly compared the effects of *Ppm1d* and *Trp53* mutations on HSC fitness. To compare how *Ppm1d* activation and *Trp53* inactivation affect the DDR, we transplanted a 1:1 mixture of bone marrow cells from *Ppm1d*^*T476∗-fl/+*^ and *Trp53*^*R172H-fl/+*^ mice. The recipients were then treated with vehicle control, cisplatin, or 2.5 Gy irradiation (Figure 3A). In the vehicle control, there was a nonsignificant trend in the peripheral blood toward *Ppm1d*^*T476∗-fl/+*^ cells having a competitive advantage and a significant difference observed in the HSC and multipotent progenitor pools (Figure 3B-C), consistent with human genetic data suggesting that *PPM1D* mutant blood cells expand more rapidly than *TP53* mutant cells in an aging population.^27^^,^^28^ In contrast, the *Trp53*^*R172H-fl/+*^ cells outcompeted the *Ppm1d*^*T476∗-fl/+*^ cells after either cisplatin or radiation exposure, with significant differences observed in the radiation group (Figure 3D-G). However, in contrast to prior data showing complete selection of *Trp53*^*R172H-fl/+*^ cells over WT cells after 2.5 Gy irradiation, the *Trp53*^*R172H-fl/+*^ cells did not fully outcompete the *Ppm1d*^*T476∗-fl/+*^ cells in this setting.^15^ These data show that Ppm1d activation suppresses the DDR but to a lesser degree than direct p53 inactivation.

### Ppm1d loss sensitizes primary leukemia cells to clinically used cytotoxic agents

**Figure 4.**
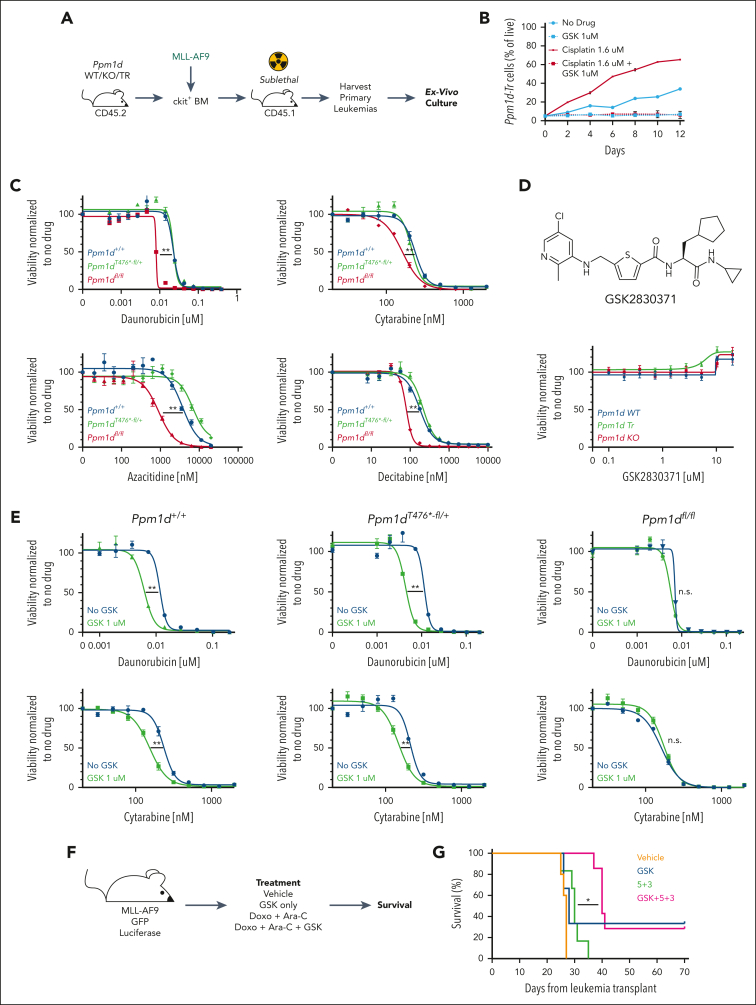
***Ppm1d* mediates sensitivity of primary leukemia cells to cytotoxic agents.** (A) Schematic of generation of primary leukemia cells using viral transduction of *MLL-AF9-GFP* into c-kit-enriched bone marrow from *Ppm1d*^*+/+*^;*Vav-Cre*^+^ (WT), *Ppm1d*^*fl/fl*^;*Vav-Cre*^+^ (KO), or *Ppm1d*^*T476*^^*∗*^^*-fl/+*^;*Vav-Cre*^+^ (TR) mice and transplantation into sublethally irradiated recipients. (B) Frequency of *Ppm1d*^*T476*^^*∗*^^*-fl/+*^ leukemia cells when grown with *Ppm1d*^*+/+*^ leukemia cells in vitro over a 10-day period in the presence of dimethyl sulfoxide (DMSO) (no drug), GSK2830371, Cisplatin, or Cisplatin and GSK2830371 (supplemental Figure 4A). (C-E) Viability of primary leukemia cells, as assessed using CellTiterGlo, after 3 days of in vitro exposure to cytotoxic therapies (B), GSK2830371 (C), or both (D). Representative figures from one of the biological replicates is shown here. (F-G) Schematic (F) and survival (G) of mice carrying *MLL-AF9*^*+*^ leukemias treated with vehicle, GSK283071, doxorubicin with Ara-C (“5 + 3”), or doxorubicin with Ara-C and GSK2830371 (“5 + 3 + GSK”). Error bars show SEM, ∗*P* < .01, ∗∗*P* < .001.

The role of Ppm1d in response to DNA damage would suggest that the loss of Ppm1d modulates the response to cytotoxic chemotherapy or radiation. We tested this hypothesis on primary leukemia cells using our engineered mouse models. First, we transduced c-kit^+^ bone marrow cells from *Ppm1d*^*+/+*^, *Ppm1d*^*T476∗-fl/+*^, or *Ppm1d*^*fl/fl*^ mice with retrovirus expressing *MLL-AF9* and green fluorescent protein (GFP), then transplanted the cells into sublethally irradiated recipients.^10^ After 8 to 12 weeks, the recipient mice developed GFP^+^ leukemia, which we isolated from the bone marrow and adapted to in vitro culture using cytokine-supplemented media (Figure 4A).

To test the relative sensitivity of leukemia cells with Ppm1d activation to cytotoxic therapies, we mixed *Ppm1d*^*T476∗-fl/+*^ leukemia cells with *Ppm1d*^*+/+*^ leukemia cells, and cultured the cells for 10 days in the presence of dimethyl sulfoxide, Cisplatin, GSK2830371 (a PPM1D inhibitor), or Cisplatin with GSK2830371 (supplemental Figure 4A).^16^^,^^29^ The *Ppm1d*^*T476∗-fl/+*^ cells displayed a moderate competitive advantage at baseline and a strong competitive advantage in the presence of cisplatin, effects that were eliminated by the addition of GSK2830371 (Figure 4B).

In contrast, leukemia cells with *Ppm1d* loss displayed an increased sensitivity to agents commonly used in the treatment of myeloid neoplasia including daunorubicin, cytarabine, decitabine, and azacitidine (Figure 4C). Pharmacologic inhibition of Ppm1d using GSK2830371 alone did not impair leukemia cell growth, but GSK2830371 synergized with daunorubicin, cytarabine, decitabine, azacitidine, and radiation to kill both *Ppm1d*^*T476∗-fl/+*^ and *Ppm1d*^*+/+*^ cells but not *Ppm1d* knockout cells (Figure 4D-E; supplemental Figure 4B).^16^^,^^29^ Similar synergistic activity of GSK2830371 was also observed with platinum salts, topoisomerase inhibitors, and, to a lesser extent, vincristine (supplemental Figure 4C).

We also assessed the effects of PPM1D inhibition on previously reported human acute myeloid leukemia (AML) PDX models.^12^^,^^13^ First, we exposed 4 different PDXs to daunorubicin or cytarabine, with and without concurrent GSK2830371 for 72 hours in culture. We found that the addition of GSK2830371 increased the sensitivity of these cells to daunorubicin and cytarabine, particularly in the *TP53* WT models (supplemental Figure 5A). Next, we tested whether GSK2830371 enhanced the mitochondrial priming of 5 separate PDX models as assessed by BH3 profiling.^14^ We found that 3 of the 5 PDXs had an average of at least 15% priming upon exposure to GSK283071, a level that has been shown to correlate to chemotherapy sensitization (supplemental Figure 5B).^14^

To examine the effect of Ppm1d inhibition on leukemia therapy in vivo, we generated murine MLL-AF9^+^ leukemias that coexpress GFP and luciferase.^11^ We confirmed leukemia cell engraftment and equal disease burden of secondary, nonirradiated recipients using bioluminescent imaging before the initiation of 4 treatment groups: vehicle, GSK2830371, cytarabine for 5 days and doxorubicin for 3 days (5 + 3), or GSK2830371 with 5 + 3 (Figure 4F; supplemental Figure 4D). As expected, the mice in the 5 + 3 group showed a prolonged survival (median, 30 vs 27 days; *P* = .02) relative to vehicle. Although there was no survival difference between the GSK2830371 and vehicle groups, one of the mice treated with only GSK2830371 had a durable response. Consistent with our in vitro data, the addition of GSK2830371 to 5 + 3 resulted in a significant prolongation of survival (median survival of 40 vs 30 days; *P* < .01), with 2 mice showing a durable response (Figure 4G). Taken together, these data suggests that PPM1D is a critical regulator of cytotoxic resistance in leukemia cells and inhibition of PPM1D, even in the absence of a *PPM1D* activating mutation, enhances the effects of cytotoxic therapy.

### *TP53* inactivation mediates resistance to PPM1D inhibition

Prior data from our group and others suggest that resistance to PPM1D inhibition is mediated by p53.^6^^,^^22^ To interrogate this association further, we analyzed gene expression and genome-wide CRISPR/Cas9 screening data from over 1000 cell lines included in the Cancer Dependency Map.^21^ Across all of the cell lines, average *PPM1D* RNA expression was higher in *TP53* WT cells, consistent with the *PPM1D* gene being a direct transcriptional target of p53 (supplemental Figure 6A).^30^ We analyzed the correlation between the activity of sgRNAs targeting *PPM1D* and all other genes. The most positively correlated genes with *PPM1D* were *MDM2* and *MDM4* (Pearson correlations 0.67 and 0.64, respectively), whereas the most negatively genes correlated were *TP53*, *TP53BP1*, and *CHEK2* (Pearson correlations −0.64, −0.55, and −0.53, respectively), confirming that the influence of PPM1D on cellular viability in these screens acts through the DDR and p53 (supplemental Figure 6B-C). Notably, these effects, including the effects of *PPM1D* knockout on cell viability, were dependent on the mutation status of *TP53*. Higher *PPM1D* expression was associated with decreased viability after *PPM1D* knockout, more so in *TP53* WT than in *TP53* mutant cells (linear regression slope −0.11 vs −0.036, respectively) (supplemental Figure 6D).

**Figure 5.**
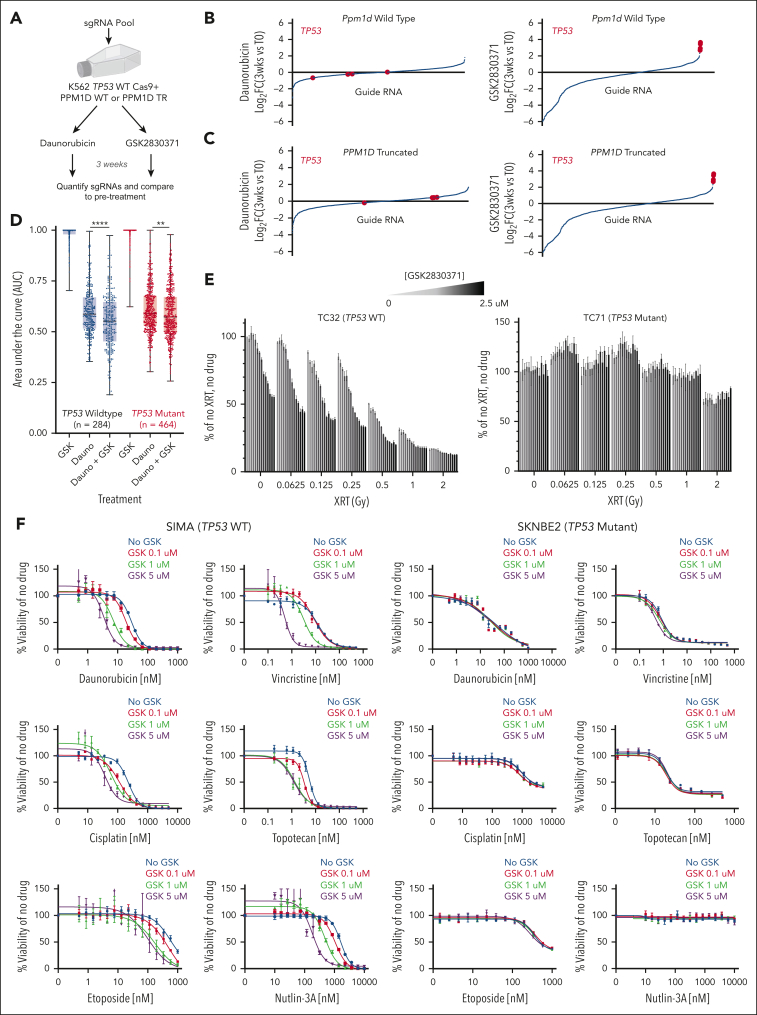
**Sensitivity to PPM1D inhibition is regulated by p53.** (A) Schematic of CRISPR/Cas9 knockout screen to assess effects of genetic knockout on sensitivity of K562 *PPM1D*-WT or *PPM1D*-truncated (TR) cells to daunorubicin or GSK2830371 over a 3-week period. (B-C) Changes in guide RNAs over experiment in *PPM1D*-WT (B) or *PPM1D*-TR (C) cells treated with daunorubicin (left) or GSK2830371 (right). Guide RNAs targeting *TP53* are highlighted in red. (D) Area under the curve (AUC) calculations for *TP53*-WT (black) or *TP53*-mutant (red) cells lines treated with either GSK2830371, daunorubicin, or daunorubicin with GSK2830371 using the PRISM platform (refer to “Methods”). (E) Viability of TC32 (left) or TC71 (right) Ewing sarcoma cells after exposure to radiation and varying doses of GSK2830371. (F) Viability of SIMA (left) or SKNBE2 (right) neuroblastoma cells after exposure to cytotoxic agents and varying doses of GSK2830371.

To identify mediators of PPM1D inhibition, we performed a pooled CRISPR/Cas9 viability screen. We introduced a truncating mutation in the C-terminus of *PPM1D* (“*PPM1D* TR”) in a previously described K562 human leukemia cell line engineered to be *TP53* WT and to express Cas9.^15^ The *PPM1D* WT and TR cells were infected with a custom pool of sgRNAs targeting genes involved in the DDR, inflammation, and P38 pathway and then grown in the presence of daunorubicin or GSK2830371 (Figure 5A). In both *PPM1D* WT and *PPM1D* TR cells, sgRNAs targeted *TP53* were the most highly selected sgRNAs across the entire library after exposure to GSK2830371 but not after culture in daunorubicin (Figure 5B-C). Thus, *TP53* loss is the strongest mechanism of resistance to PPM1D inhibition, regardless of the presence of an activating mutation.

### PPM1D inhibition augments cytotoxic agents across many tissue types

Previous studies have shown that GSK2830371 inhibits growth of several cell lines. To examine this more systematically and determine whether PPM1D inhibition could be a viable strategy to sensitive nonhematopoietic malignancies to cytotoxic therapy, we performed a large-scale cell line viability screen. Using the previously described PRISM platform, we performed a drug sensitivity screen across 748 cells lines using 8-point dose responses of GSK2830371 alone, daunorubicin alone, or daunorubicin with GSK2830371.^20^

Consistent with our prior data, monotherapy with GSK2830371 was active in very few cell lines, whereas the addition of GSK2830371 significantly enhanced daunorubicin-induced toxicity, particularly in *TP53* WT cell lines (Figure 5D). Indeed, 67% (31/46) of cell lines that were sensitized to daunorubicin-induced toxicity by GSK2830371 were *TP53* WT, compared with 23% (284/748) of all cell lines screened. Among the 31 *TP53* WT cell lines, we noted a high frequency of mesenchymal origin, particularly of bone or soft tissue (13/31).

Based on these findings, we explored the impact of PPM1D inhibition using Ewing sarcoma and neuroblastoma cell line models, because both tumors are often *TP53* WT and clinically treated with DNA damaging agents including chemotherapy and radiation. We first compared the effect of GSK2830371 on sensitization to radiation in 2 Ewing sarcoma (EWS) lines: TC32, which is *TP53* WT, and TC71, which is *TP53* mutant. Cells were treated with varying doses of GSK2830371 and radiation, then viability was analyzed 3 days later. We found that at all doses of radiation the TC32 cells, but not the TC71 cells, were sensitized with increasing doses of GSK2830371 (Figure 5E). Similarly, in the neuroblastoma (NB) context, *TP53* WT SIMA cells were sensitized by GSK2830371 to inducers of the DDR, including Nutlin-3a, but this was not observed in the *TP53*-mutant SKNB2 line (Figure 5F). These results demonstrate that PPM1D renders *TP53* WT cells with more resistance to genotoxic stresses and pharmacologic inhibition of PPM1D can enhance the activity of cytotoxic agents.

## Discussion

We developed conditional mouse models of *Ppm1d* truncation and *Ppm1d* deletion and found that *Ppm1d* truncation increases HSC fitness at baseline and in the presence of genotoxic stress and enhances the ability of HSCs to serially transplant. We further found that primary leukemia cells use Ppm1d to attenuate the cytotoxic effects of clinically used therapies and that genetic loss or pharmacologic inhibition of Ppm1d sensitizes mouse and human leukemia cells to these agents in vitro and in vivo. In contrast, acute loss of *Ppm1d* in adulthood throughout the entire organism was tolerated with minimal observed toxicity. These data support PPM1D inhibition, particularly in combination with radiation or chemotherapy, as a therapeutic strategy.

Our mouse models enabled us to examine the effect of genetic or pharmacologic loss on leukemia cells. Genetic loss or pharmacologic inactivation of *Ppm1d* rendered primary leukemia cells more sensitive to the cytotoxic therapies used for AML, whereas activation of *Ppm1d* conferred a resistance phenotype. In vivo studies demonstrated that the addition of GSK2830371 to chemotherapy prolonged the survival of mice that received transplantation with a highly aggressive leukemia. These data suggest that inhibition of PPM1D may provide therapeutic value when added to cytotoxic therapies, independent of the presence of an activating *PPM1D* mutation. More broadly, we found that PPM1D inhibition sensitizes cells to both chemotherapy and radiation.

To examine the toxicity of *Ppm1d* inhibition, we deleted *Ppm1d* throughout the adult mouse and found little toxicity. Aside from moderately impaired lymphopoiesis, a previously described phenomenon in the *Ppm1d* germ line knockout animals, we did not observe a significant effect of *Ppm1d* activation or deletion, either early in development or in adulthood, on hematopoiesis at baseline.^8^ Importantly, organism-wide loss of *Ppm1d* induced at age 10 weeks did not have any observable deleterious effects on the mice, even after an irradiation insult. We did not observe the variable male runting, reproductive organ atrophy, or altered male longevity seen in the germ line knockout, likely because we induced *Ppm1d* deletion in the postnatal setting.^8^ Our data indicate that inhibition of PPM1D may be well tolerated, and notably, it does not cause thrombocytopenia, a common toxicity associated with other modulators of the DDR, including the nutlin class of drugs.^31^

We found that a conditional *Ppm1d* activating mutation enhanced the competitive fitness of HSCs and increased the ability of HSCs to serially transplant. We observed a more potent selective effect with radiation compared with cisplatin, which may be related to either the mechanism and degree of DNA damage or the dosing of the drug. This result contrasts with the work by *Hsu et al*, in which hematopoietic cells carrying a germ line *Ppm1d*^*R451X*^ alteration did not show a competitive advantage in the absence of cytotoxic therapy but did display impaired serial transplantation.^6^ This discrepancy could be because of the difference in the site of the mutation (R451 vs T476), the difference between a germ line alteration and conditional allele, minor differences in mouse background strains, or differences in vivarium. Our findings are consistent with human genetic data showing that clonal, somatic *PPM1D* activating mutations in hematopoietic cells are often observed in patients without a history of prior cytotoxic exposure, albeit at a lower frequency than that observed in cohorts with such exposures. In the former cases, the HSCs carrying *PPM1D* mutations expand over time in the absence of known exogenous stresses and are sometimes present at a young age.^1^^,^^27^^,^^32^^,^^33^

We probed the relationship between *TP53* and *PPM1D* mutations in HSCs using our models. Somatic, clonal hematopoietic mutations in both genes are commonly identified in patients treated with cytotoxic therapy. We found that in the absence of an exogenous stress, there is no selection of 1 mutation over the other; whereas a heterozygous *Trp53* mutation (the allelic state often observed in clonal hematopoiesis) confers a stronger fitness advantage to cells than a truncating *Ppm1d* mutations after exposure to cytotoxic therapy. This is consistent with human data suggesting that the variant allele fraction of *TP53* mutations is often higher than that for *PPM1D* when found in the same patient who has a cytotoxic exposure history.^26^ These data indicate that although PPM1D is able to dephosphorylate and decrease activity of p53 and other proteins upstream and downstream of p53 in the DDR pathway, ultimately, the loss of p53 is likely a more potent suppressor of the DDR.

To probe the dependence of PPM1D activity on p53, we performed a CRISPR/Cas9 resistance screen in a human AML cell line and found that inhibition of PPM1D by GSK2830371 resulted in strong selection of sgRNAs targeting *TP53*, suggesting that PPM1D inhibition requires p53 for effects on cellular proliferation. To extend this finding beyond leukemia, we reanalyzed the Cancer Dependency Map and confirmed that the proliferative effects of *PPM1D* knockout were dependent of the cellular *TP53* mutation status. Using a multiplexed screening system of 748 cell lines, we again found that the degree to which PPM1D inhibition with GSK280371 sensitized cells to daunorubicin was also *TP53*-dependent and confirmed these results in 2 distinct cellular contexts, Ewing sarcoma and neuroblastoma. Although these data strongly support the role of p53 in mediating PPM1D biology in the context of cellular proliferation and response to cytotoxic therapy, they do not preclude the possibility that other, p53-independent pathways, are also relevant to PPM1D biology in similar or distinct cellular contexts. These data support the use of PPM1D inhibition as a therapeutic strategy in *TP53* WT cancers and indicate that *TP53* mutations may emerge as a mechanism of resistance to this approach.

This study highlights the important roles that PPM1D plays in normal and malignant hematopoiesis while further elucidating genetic observations from human cohorts. Our chemo-sensitization and toxicity data suggest that PPM1D inhibition may allow for effective suppression of the DDR while avoiding excessive toxicity and provides a framework and foundation for pursuing PPM1D as a therapeutic target across many oncologic contexts.

Conflict-of-interest disclosure: P.G.M. reports consulting fees from Foundation Medicine and Roche; A.S.S. reports consulting fees from Adaptive Technologies and Roche. M.S. has received research funding from Calico Life Sciences LLC. J.K. receives employment income from Third Rock Ventures. K.S. receives grant funding from the Dana Farber Cancer Institute/Novartis Drug Discovery Program and KronosBio; is a member of the scientific advisory board and has stock options with Auron Therapeutics; and has consulted for AstraZeneca. B.L.E. has received research funding from 10.13039/100006436Celgene and Deerfield Ventures, consulting fees from GRAIL, and is on the scientific advisory boards for Exo Therapeutics and Skyhawk Therapeutics. The remaining authors declare no competing financial interests.
