# Supplementary material for: PPM1D modulates hematopoietic cell fitness and response to DNA damage and is a therapeutic target in myeloid malignancy
Source: Blood. 2023 Aug 20;142(24):2079–91. doi: 10.1182/blood.2023020331 (PMC10733824; doi:10.1182/blood.2023020331)
Supplement: Supplemental Methods and Figures [file BLOOD_BLD-2023-020331-mmc1.pdf]

SUPPLEMENTAL MATERIAL

Supplemental Methods………………………………………………………………...…..2-4

Supplemental Figures S1-S6…………………………………………………………...…5-10

SUPPLEMENTAL METHODS

Inducible Mouse Models

The mice were then crossed to one of the following lines: *Vav-Cre*, *Mx-Cre*, or *Cre-ERT2*. Induction of Cre-recombinase in the *Mx-Cre* background was achieved by intraperitoneal administration of two doses of 250 ug of poly(I:C) (pIpC).[9,10] Induction of Cre-recombinase in the *Cre-ERT2* background was achieved by intraperitoneal administration of tamoxifen at a dose of 75 mg/kg daily for five days.

Competitive Bone Marrow Transplantation and Cytotoxic Treatments

Whole bone marrow cells from $Ppm1d^{+/+}$*;Vav-Cre*$^{+}$*;CD45.2* or $Ppm1d^{T476\text{-}fl/fl}$*;Vav-Cre*$^{+}$*;CD45.2* or $Ppm1d^{fl/fl}$*;Vav-Cre*$^{+}$*;CD45.2* were mixed 1:1 with whole bone marrow cells from *Vav-Cre*$^{+}$*;CD45.1/2* mice in phosphate buffer saline and retro-orbitally injected into lethally irradiated (split dose of 450 cGy x 2) Bl6.SJL *CD45.1*$^{+}$ recipient mice. Separately, whole bone marrow cells from $Ppm1d^{T476\text{-}fl/fl}$*;Vav-Cre*$^{+}$*;CD45.2* and $Trp53^{R172H/+}$*;Vav-Cre*$^{+}$*;CD45.1/2* were mixed 1:1 and retro-orbitally injected into lethally irradiated (split dose of 450 cGy x 2) Bl6.SJL *CD45.1*$^{+}$ recipient mice. Treatment of the recipient mice started at four weeks after transplant and included All serial transplantations were performed on the indicated animals using whole bone marrow retro-orbitally injected into lethally irradiated Bl6.SJL *CD45.1*$^{+}$ recipient mice.

Peripheral Blood and Bone Marrow Analyses

Peripheral blood was isolated at the indicated timepoint by retro-orbital collection and subject to RBC lysis (Qiagen). Blood counts were performed on a Hemavet (Drew Scientific) and peripheral blood chimerism was assessed using flow cytometry (BD FACSCanto II) using the following antibodies (Biolegend): anti-CD3-PE-Cy7, anti-B220-BV510, anti-Gr1-PacBlue, anti-CD11b-APC-Cy7, anti-CD45.1-FITC, anti-CD45.2-PE. Bone marrow stem and progenitor cell analysis was performed as follows. Following euthanasia, the femur, tibia, pelvis, and spine were isolated from the indicated mice, crushed, filtered and subject to RBC lysis (Qiagen). Single cell suspensions were stained and analyzed by flow cytometry on the BD FACSCanto II.

For the stem cell analysis in non-transplanted mice the following antibodies (Biolegend) were used: anti-CD11b-PerCP-Cy5, anti-GR-1-PerCP-Cy5, anti-Ter-119-PerCP-Cy5, anti-B220-PerCP-Cy5, anti-CD3-PerCP-Cy5, anti-Kit (2B8)-APC, anti-Sca1-PE-Cy7, anti-CD150-PE, anti-CD48-APC-Cy7, anti-Flk2-BV421 (PB), anti-CD16/32-BV510, and anti-CD34-FITC. For progenitor cell analysis in non-transplanted mice the following antibodies (Biolegend) were used: anti-CD11b-PerCP-Cy5, anti-GR-1-PerCP-Cy5, anti-B220-PerCP-Cy5, anti-CD3-PerCP-Cy5, anti-Kit (2B8)-APC, anti-Sca1-PE-Cy7, anti-CD150-PE, anti-CD16/32-BV510, anti-CD41-ApcCy7, anti-CD105-PacBlue, and anti-Ter119-FITC.

For the stem cell analysis in the transplanted mice the following antibodies (Biolegend) were used: anti-Ter119-PeCy5, anti-B220-PeCy5, anti-CD3-PeCy5, anti-Gr1-PeCy5, anti-CD11b-PeCy5, anti-cKit-APC, anti-Sca1-PE-Cy7, anti-CD48-Apc-Cy7, anti-CD150-PE, anti-CD45.1-FITC, and anti-CD45.2-PacBlue. For the progenitor cell analysis in the transplanted mice the following antibodies (Biolegend) were used: anti-Ter119-PeCy5, anti-B220-PeCy5, anti-CD3-

PeCy5, anti-Gr1-PeCy5, anti-CD11b-PeCy5, anti-cKit-APC, anti-Sca1-PE-Cy7, anti-CD34-FITC, anti-CD16/32-BV510, anti-CD45.1-PE, and anti-CD45.2-PacBlue.

In Vitro Drug Exposure of Mouse Leukemia Cells

In vitro exposure experiments were performed as follows. For the three-day viability analyses, each genotype, 5,000 cells were plated in 50 ul of media per well of 384-well plates. The indicated drugs (Selleck Chemicals) were diluted in DMSO and added to the appropriate wells o using an automated drug dispenser (Tecan) or the plates were exposed to the indicated dose of radiation. Three days later, the 10 ul of CellTiter-Glo (Promega) were added to each well and the luminescence signal was measured using an EnVision plate reader (PerkinElmer).

For the *in vitro* resistance experiments, *RFP657*$^{+}$ *Ppm1d*$^{T476\text{-}fl/fl}$ and *BFP*$^{+}$ *Ppm1d*$^{+/+}$ leukemia cells were mixed in a 1:20 ratio then plated at 50,000 cells per well in 200 ul of media into a 96 well plate. The indicated dose of drugs (Selleck Chemicals) were added to each well using the automated drug dispenser (Tecan). Every 3-4 days the cells were replated in fresh media and analyzed for the ratio of RFP657:BFP using a BD FACSCanto II.

In Vitro Drug Exposure of Human Patient Derived Xenograft Models

Primary patient samples were acquired following written informed consent per the Declaration of Helsinki, and patient-derived xenografts (PDX) models were established under protocols approved by Dana-Farber Cancer Institute and Cincinnati Children's Hospital Medical Center institutional review boards. For short-term in vitro culture, PDX cells were maintained in IMDM containing 20% FBS and 1% PS and supplemented with 10 ng/mL human SCF, TPO, FLT3L, IL3, and IL6 (PeproTech 300-07, 300-18, 300-19, 200-03 and 200-06). Cells were exposed to drugs at the indicated concentrations for 72 hours and viability was measured using the CellTiter-Glo reagent.

Dynamic BH3 Profiling of Human Patient Derived Xenograft Models

All animal studies were performed in accordance with approved IACUC guidelines at Dana-Farber Cancer Institute animal facility (IACUC protocol #14-038) and at National University of Singapore facility (IACUC protocol # R20-0342). AML PDX models are available from the Center for Patient-Derived Models at Dana-Farber Cancer Institute (https://www.pdxfinder.org/source/dfci-cpdm/). Female NSG mice at 6-8 weeks of age (Jackson Labs) were injected with 0.6 million human leukemia cells intravenously (IV). Following transplant, mice were bled weekly, and treatment was initiated when circulating leukemia burden was >5% as assessed by flow cytometry staining for hCD45 (clone HI30, BD Biosciences) and hCD33 (clone WM53, BD Biosciences). Animals were humanely euthanized, and the spleen and bone marrow were processed into single cell suspensions. Red blood cell lysed cells were plated with heat-inactivated RPMI-1640 (Invitrogen) and 10% fetal bovine serum in 24-well plates at 1 million/mL and exposed to a GSK compound for 14 h or DMSO (control) followed by BH3 profiling (Ryan et al., 2016). Briefly, cells were exposed to synthetic BH3 peptides after plasma membrane permeabilization by 5% digitonin. The gating strategy included antibody markers for CD45 (Fisher Scientific, Clone HI30), CD34 (BioLegend, Clone 581), CD38 (BioLegend, Clone HIT2), CD33 (Fisher Scientific, Clone WM53), c-kit (BioLegend,

Clone 104D2), and live/dead fixable zombie yellow stain (BioLegend). AML blasts were identified by CD45 lo-mid / SCC-low. Sensitivity to BH3 peptides was measured as percent cytochrome c release (BioLegend, 6H2.B4) loss as determined by FACS. DMSO was used as a negative control for cytochrome c release, whereas a control without the cytochrome c antibody was used as a positive control for 100% cytochrome c release. The read-out of dynamic BH3 profiling is drug-induced change in priming defined as "delta priming" (calculation: delta priming = cytochrome c loss[drug] - cytochrome c loss[DMSO]). An acceptable delta priming threshold (<15%) calculated by cytochrome c release caused by 3(mean ± SD) of DMSO treated wells was used to determine significance.

CRISPR/Cas9 Screen

Human K562 cells were grown in RPMI (Gibco) supplemented with 10% FBS. K562 cells were engineered to carry a wild-type copy of *TP53* and a truncated version of *PPM1D* as previously described.[12,13] The cells were infected with a custom library of sgRNAs encoded by lentivirus obtained from the Broad Institute. Two days after infection the cells were selected in 2 ug/ml puromycin for three days then cultured for the following three weeks (media change every three days) in the presence of either DMSO (Sigma Aldrich), daunorubicin (Selleck Chemicals), or GSK2830371 (Selleck Chemicals). An aliquot of cells was isolated immediately following puromycin selection and after 21 days of culture. DNA was isolated from the cells and the relative representation of each sgRNA was quantified using next generation sequencing as previously described.[14,15]

Human Cell Line Drug Exposures

The human cell lines were cultured in the RPMI (Gibco) + 10% FBS (TC32, TC71, SIMA) or DMEM/F12 (Gibco) +10% FBS (SKNBE2). For the drug or radiation treatments, 5,000 cells were plated in 50 ul of media per well of 384-well plates. The indicated drugs (Selleck Chemicals) were diluted in DMSO and added to the appropriate wells o using an automated drug dispenser (Tecan) or the plates were exposed to the indicated dose of radiation. Three days later, the 10 ul of CellTiter-Glo (Promega) were added to each well and the luminescence signal was measured using an EnVision plate reader (PerkinElmer).

PRISM Screen and Analysis of Cancer Dependency Map Data

750 DNA-barcoded cell lines grown in pools of approximately 100 cell lines per pool were exposed for five days to 8 different doses of daunorubicin (range from 0.1nM to 2.5 uM), 8 doses of GSK2830371 (range from 1nM to 10 uM), or 8 doses of daunorubicin (range from 0.1nM to 2.5 uM) with 2.5 uM GSK2830371. The relative viability of each treatment was assessed by comparing the representation of each cell line, assessed by quantification of DNA barcodes, in the treated wells compared to the negative control (DMSO) and positive control (Bortezomib) wells. Using these normalized viability values, the area under the curve for each treatment condition was then calculated.[16] The publicly available 22Q4 release was used. DNA mutation data, RNA-sequencing expression data (Log_2(Total counts per million + 1), and CRISPR/Cas9 gene knockout dependencies (CERES scores) were obtained for all available cell lines and analyzed, stratified based on *TP53* mutation status, as defined as any mutation present.[19]

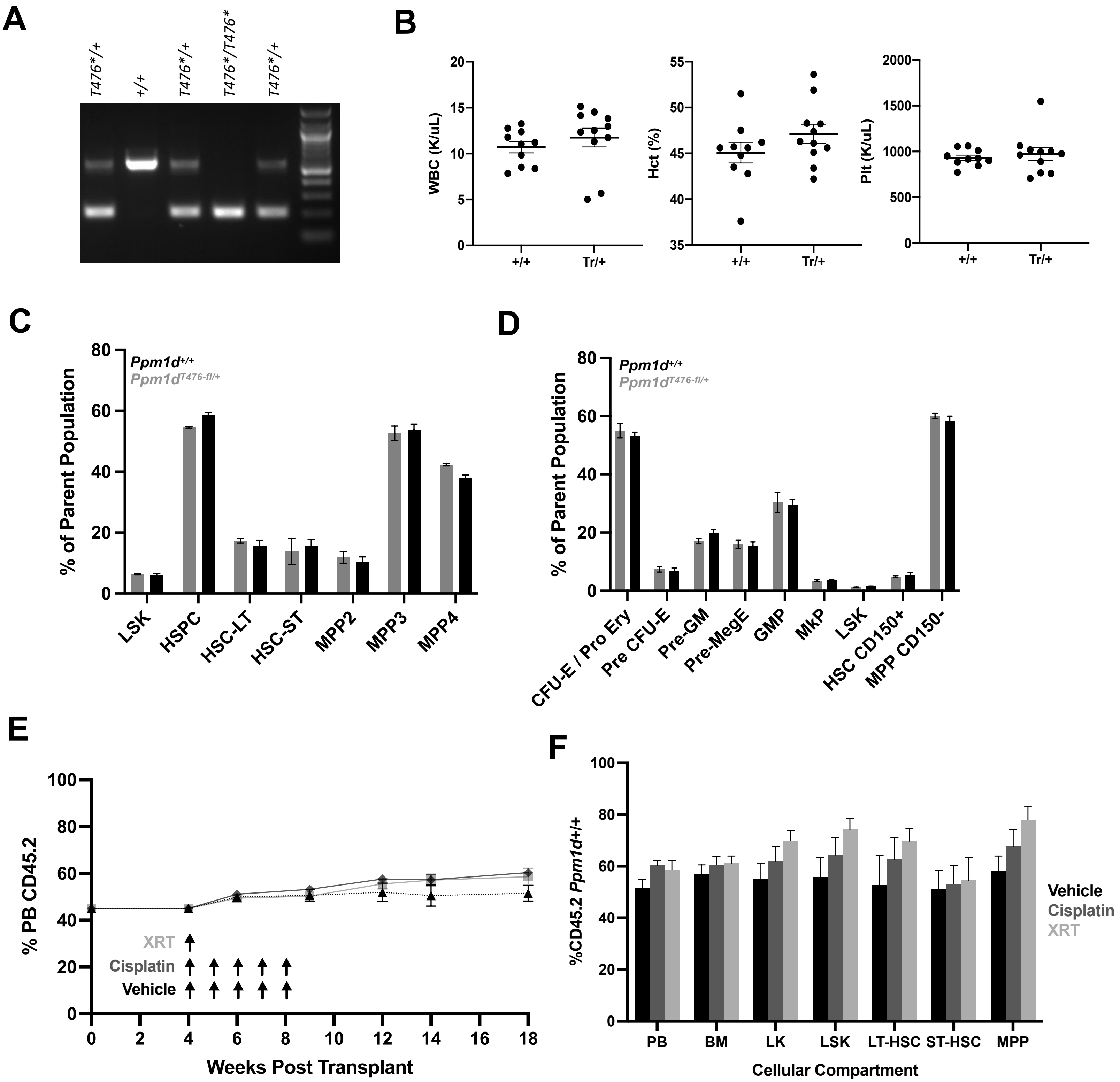

Supplemental Figure 1. Truncating mutations in *Ppm1d* enhance hematopoietic stem cell fitness.

(A) Genotyping PCR showing different allelic states of *Ppm1d*$^{T476\text{-}fl}$ mice.

(B) Peripheral blood white blood cell (WBC), hematocrit (Hct), and platelet (Plt) counts in *Ppm1d*$^{+/+}$*;Vav-Cre*$^{+}$ (+/+) and *Ppm1d*$^{T476\text{-}fl}$*;Vav-Cre*$^{+}$ (Tr/+) mice.

(C-D) Bone marrow stem and progenitor cell analysis of *Ppm1d*$^{+/+}$*;Vav-Cre*$^{+}$ and *Ppm1d*$^{T476\text{-}fl}$*;Vav-Cre*$^{+}$ mice at three months of age.

(E-F) Peripheral blood (E) and bone marrow (F) CD45.2 chimerism of recipient mice from competition experiment between *Ppm1d*$^{+/+}$*;Vav-Cre*$^{+}$*;Cd45.2* and wild-type *Cd45.1/2* competitor cells outlined in Figure 1D.

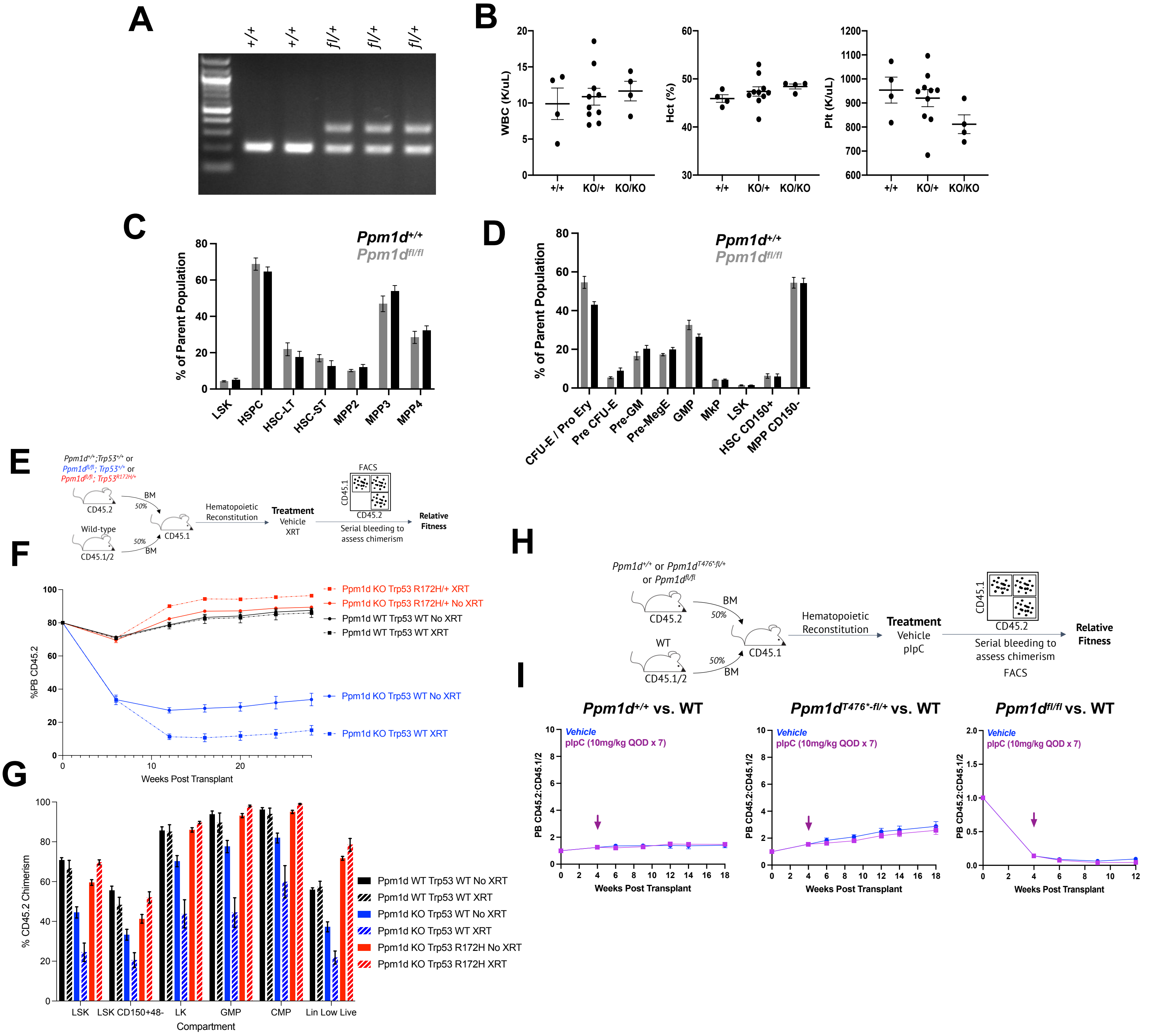

Supplemental Figure 2. *Ppm1d* loss impairs hematopoietic stem cell fitness.
(A) Genotyping PCR showing different allelic states of *Ppm1d*$^{fl/fl}$ mice.
(B) Peripheral blood white blood cell (WBC), hematocrit (Hct), and platelet (Plt) counts in *Ppm1d*$^{+/+}$*;Vav-Cre*$^{+}$ (+/+), *Ppm1d*$^{fl/+}$*;Vav-Cre*$^{+}$ (KO/+), and *Ppm1d*$^{fl/fl}$*;Vav-Cre*$^{+}$ (KO/KO) mice.
(C-D) Bone marrow stem and progenitor cell analysis of *Ppm1d*$^{+/+}$*;Vav-Cre*$^{+}$ and *Ppm1d*$^{fl/fl}$*;Vav-Cre*$^{+}$ mice at three months of age.
(E-G) Competition experiment between *Ppm1d*$^{+/+}$; *Trp53*$^{+/+}$*;Vav-Cre*$^{+}$;*Cd45.2* or *Ppm1d*$^{fl/fl}$;*Trp53*$^{+/+}$*;Vav-Cre*$^{+}$;*Cd45.2* or *Ppm1d*$^{fl/fl}$;*Trp53*$^{R172H/+}$*;Vav-Cre*$^{+}$;*Cd45.2* and wild-type *Vav-Cre*$^{+}$;*Cd45.1/2* control bone marrow cells transplanted into lethally irradiated *Cd45*.1 recipients. Sublethal irradiation was dosed at 2.5 Gy. Peripheral blood (F) and bone marrow (G) CD45.2 chimerism
(H-I) Competition experiment between *Ppm1d*$^{+/+}$*;Vav-Cre*$^{+}$;*Cd45.2* or *Ppm1d*$^{fl/fl}$;*Vav-Cre*$^{+}$;*Cd45.2* or *Ppm1d*$^{T476*/+}$*;Vav-Cre*$^{+}$;*Cd45.2* and wild-type *Vav-Cre*$^{+}$;*Cd45.1/2* control bone marrow cells transplanted into lethally irradiated *Cd45*.1 recipients. Peripheral blood CD45.2 chimerism (F) was assessed ever four weeks before and after exposure to pIpC (purple) or vehicle control (blue).

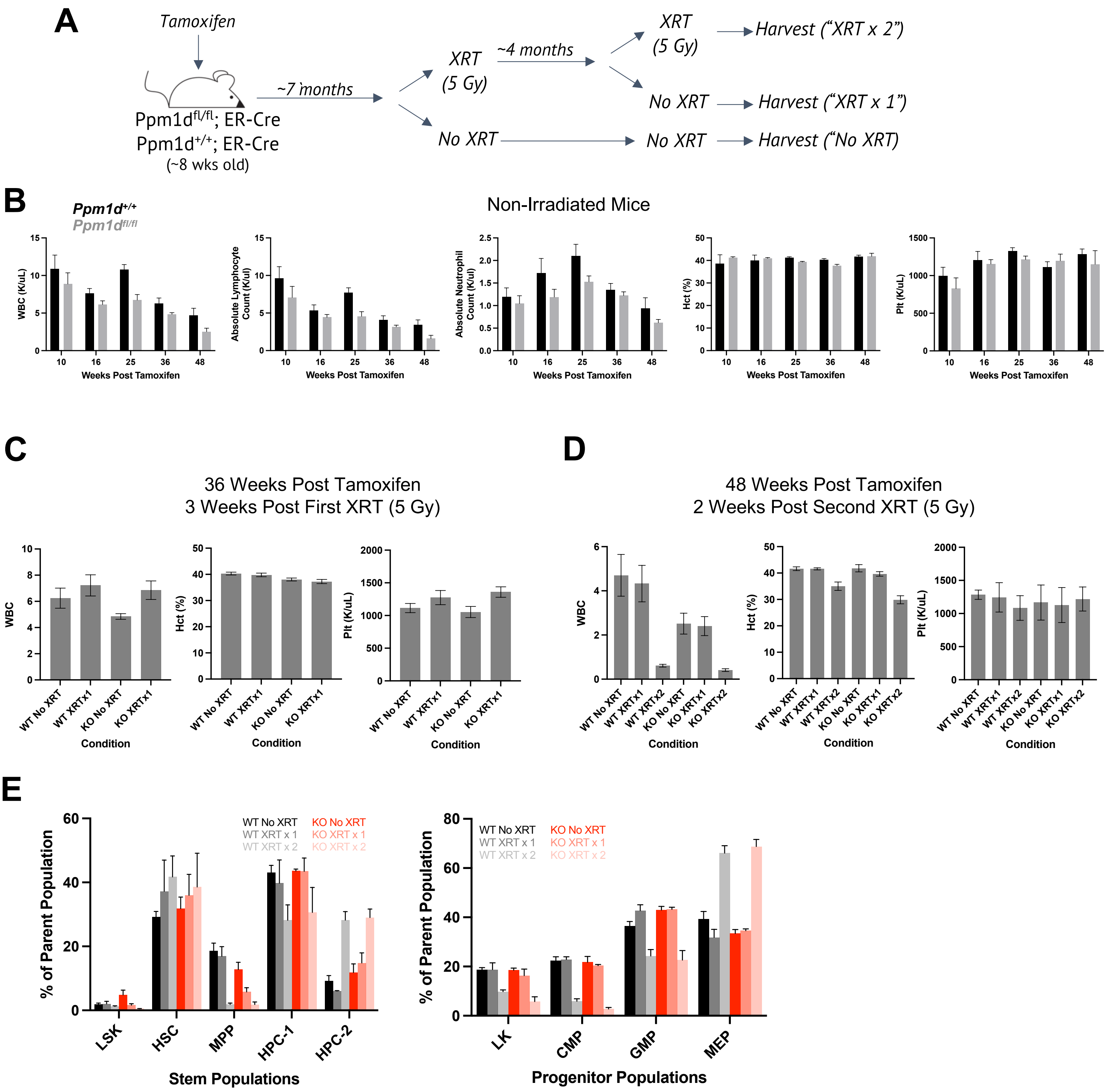

Supplemental Figure 3. Organism-wide *Ppm1d* loss in adult hood is tolerated without excessive hematologic toxicity.

(A) Schematic of experimental setup. *Ppm1d$^{fl/fl}$;Cre-ERT2^{+}* or *Ppm1d$^{+/+}$;Cre-ERT2^{+}* mice were treated with tamoxifen at 8 weeks of age then observed for 7 months. The mice were then exposed to either no radiation, a single, 5 Gy dose of radiation, or two sequential, 5 Gy doses of radiation prior to harvest and analysis.

(B) Peripheral blood counts of non-irradiatied mice.

(C-D) Peripheral blood counts of *Ppm1d$^{+/+}$;Cre-ERT2^{+}* (WT) or *Ppm1d$^{fl/fl}$;Cre-ERT2^{+}* (KO) mice 3 weeks after first dose of irradiation (C) or 2 weeks after second dose of irradiation (D).

(E) Terminal stem and progenitor bone marrow cell analysis *Ppm1d$^{+/+}$;Cre-ERT2^{+}* (WT) or *Ppm1d$^{fl/fl}$;Cre-ERT2^{+}* (KO) mice.

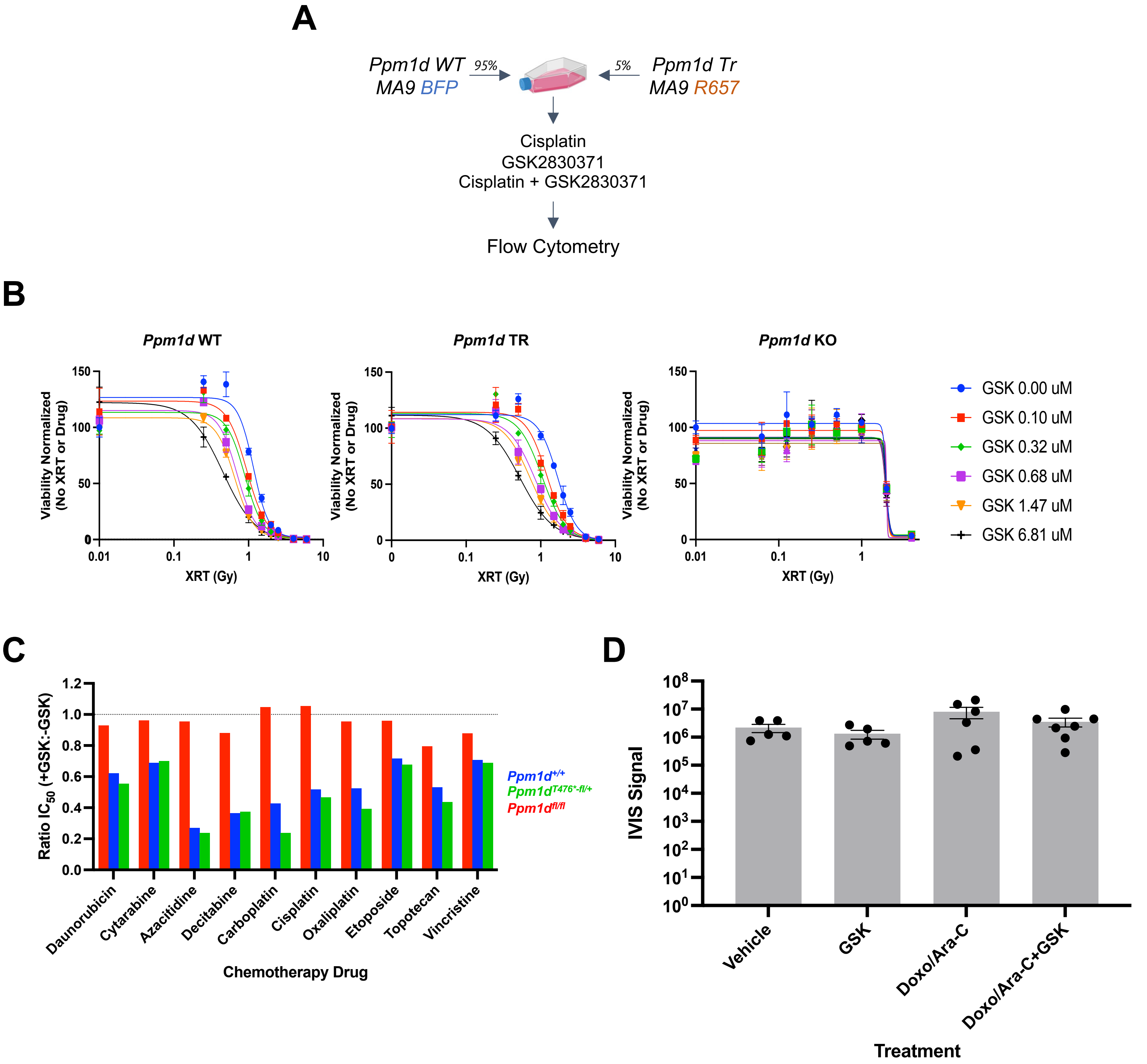

Supplemental Figure 4. Ppm1d mediates sensitivity of primary leukemia cells to cytotoxic agents.
(A) Schematic of competition experiment in which *Ppm1d*$^{+/+}$;*Vav-Cre*$^{+}$ or *Ppm1d*$^{T476\text{-}fl/+}$;*Vav-Cre*$^{+}$ leukemia cells are labeled with BFP or RFP657 then grown in a mixture while exposed to various treatments.
(B) Viability of primary leukemia cells, as assessed using CellTiterGlo, after three days of *in vitro* exposure to varying doses of radiation (XRT) and GSK2830371.
(C) Ratio of EC_{50} with or without concurrent GSK2830371 treatment for different cytotoxic drugs over three days, stratified by genotype.
(D) *In vivo* bioluminescent imaging (IVIS) signal of luciferase-expressing MLL-AF9 leukemia cells present in mice prior to initiation of treatment (see Figures 4F-G).

A

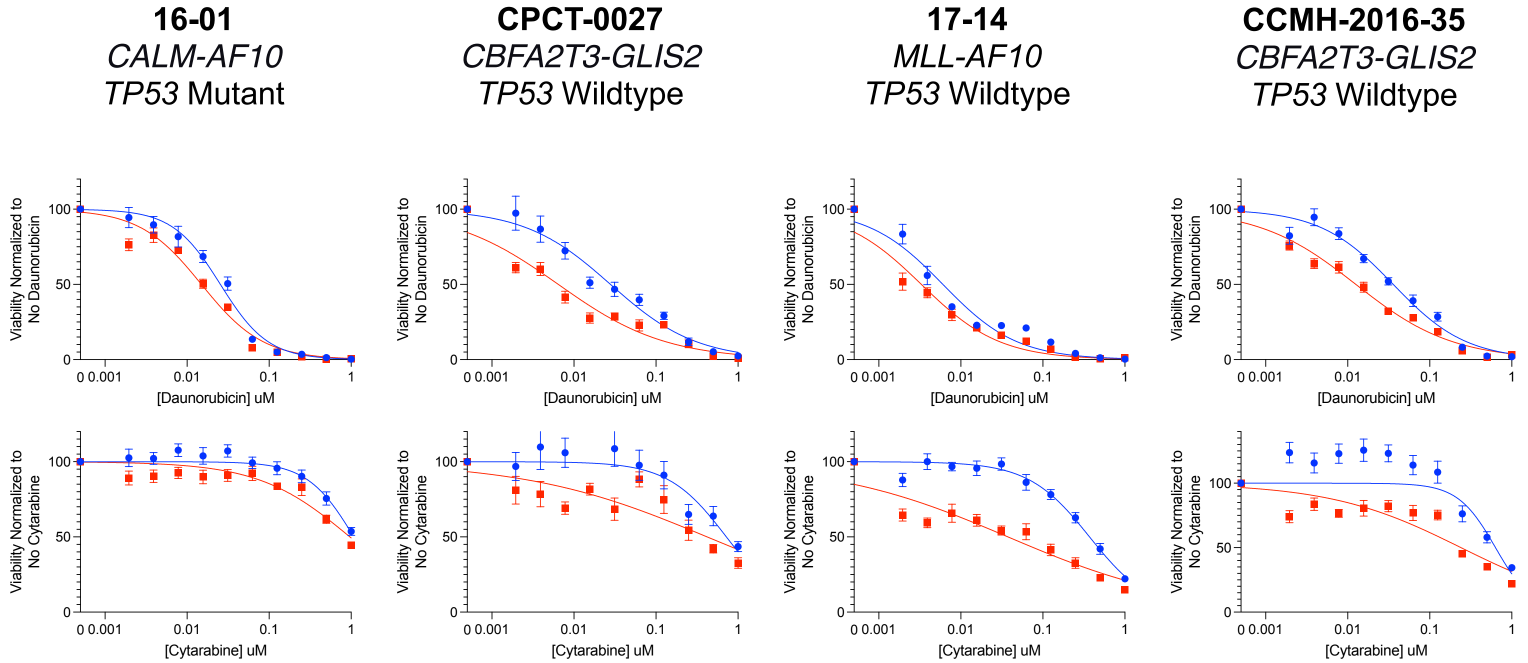

B

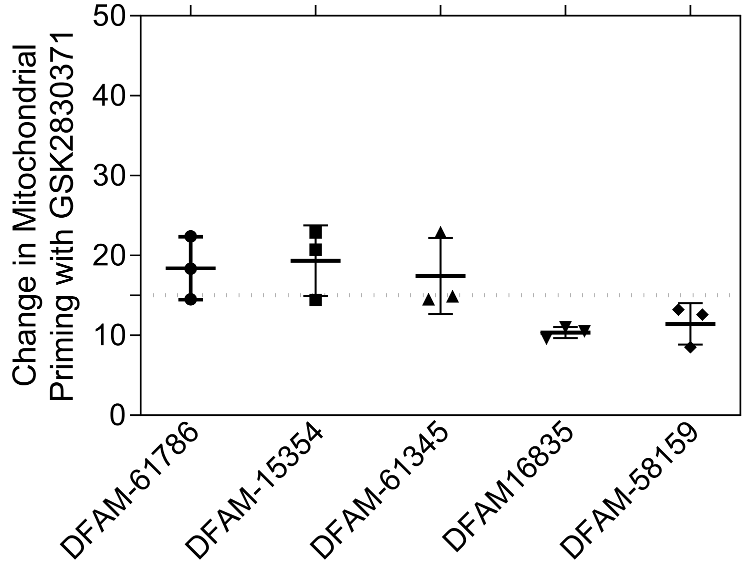

Supplemental Figure 5. Sensitivity of Human AML Cells to Pharmacologic PPM1D Inhibition.
(A) Viability of human AML patient derived xenografts (PDX), as assessed using CellTiterGlo, after three days of *in vitro* exposure to either daunorubicin or cytarabine alone (blue) or in combination with 1 uM GSK2830371 (red).
(B) Change in mitochondrial priming across five human AML PDXs after treatment with GSK2830371. Myeloblasts harvested from indicated PDX models (n=3 mice/model) were exposed to GSK2830371 for 14 hours, followed by dynamic BH3 profiling to determine delta priming in response to BIM-BH3. Plotted here is delta priming values. Delta priming = % cytochrome c release (drug treated-DMSO treated cells).

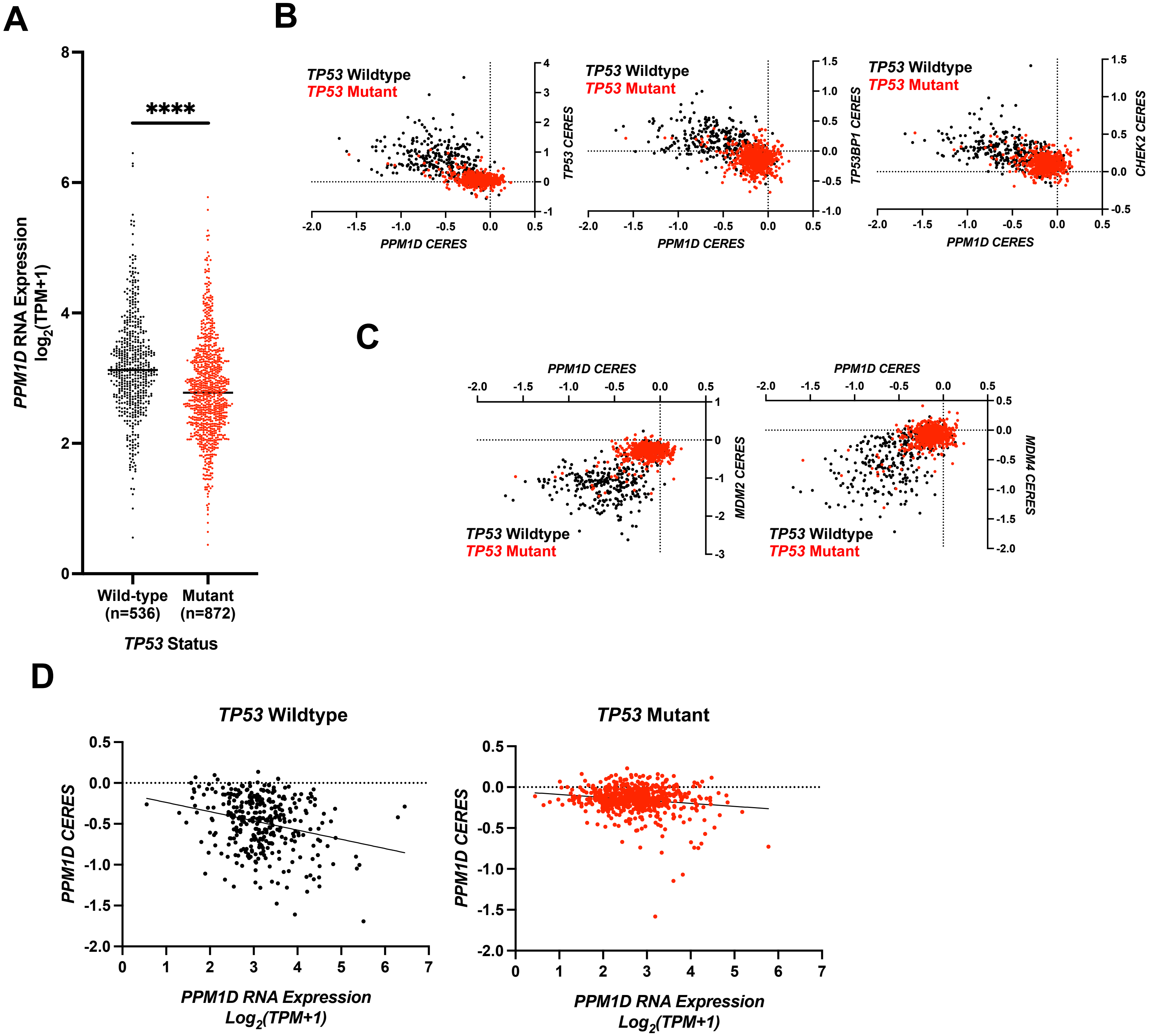

Supplemental Figure 6. Sensitivity to PPM1D inhibition is regulated by p53.

(A) RNA expression levels of PPM1D across 989 cell lines profiled as part of the Cancer Dependency Map, stratified by *TP53* mutational status. **** p-value < 0.0001

(B) Sensitivity of cell lines to CRISPR-Cas9 mediated knockout of *PPM1D* and *TP53*, as assessed by the CERES score which corrects for copy number differences between cell lines. A negative CERES score reflects impaired cell growth with knockout of the target gene. The *TP53* mutant lines are highlighted in red.

(C) Comparison of PPM1D RNA expression levels and sensitivity to PPM1D knockout, as measured by the CERES score, in *TP53* wildtype (left) and mutant (right) cell lines.
